# Curcumin-Based Photodynamic Sterilization for Preservation of Fresh-Cut Hami Melon

**DOI:** 10.3390/molecules24132374

**Published:** 2019-06-27

**Authors:** Yilin Lin, Jiamiao Hu, Shiyang Li, Siti Sarah Hamzah, Huiqin Jiang, Arong Zhou, Shaoxiao Zeng, Shaoling Lin

**Affiliations:** 1 College of Food Science, Fujian Agriculture and Forestry University, Fuzhou 350002, Fujian, China; 2 Institute for Medical Research, Jalan Pahang, 50588 Kuala Lumpur, Malaysia

**Keywords:** Hami melon, curcumin, photodynamic, colony number, storage quality

## Abstract

Fresh-cut fruits and vegetables are the main sources of foodborne illness outbreaks with implicated pathogens such as *Escherichia coli* O157:H7, *Salmonella*, and *Listeria monocytogenes*. This study aimed at investigating the influence of two key parameters (concentration of curcumin and illumination time) on the effects of curcumin-based photodynamic sterilization on the preservation of fresh-cut Hami melons. The results indicated that illumination with 50 μmol/L curcumin for 60 min using a blue LED lamp reduced the total aerobic microorganism count by ~1.8 log CFU/g in fresh-cut Hami melons. Besides this, the effects of photodynamic sterilization on the soluble solids content, color, water content, firmness, and sensory indices of the fresh-cut Hami melons were also evaluated. Compared to the control group, photodynamic sterilization can effectively delay the browning rate and maintain the luminosity, firmness, water content, and soluble solids content of fresh-cut Hami melon. The sensory quality was indeed preserved well after 9 days of storage in a fridge. These results showed that photodynamic sterilization is an effective and promising technology to prolong the shelf life of fresh-cut Hami melons.

## 1. Introduction

Fruits are an integral part of our daily diet and are rich sources of vitamins, micronutrients, and antioxidants [1]. Driven by increasing demand for convenient, ready-to-eat foods, the market of fresh-cut fruits (also known as pre-cut fruits) has grown rapidly. However, alongside this trend, foodborne disease outbreaks associated with fresh fruits have also risen globally. Recent statistics showed that the pathogens that most frequently contribute to foodborne illness outbreaks linked to fresh fruits and vegetables are *Escherichia coli O157:H7*, *Salmonella*, and *Listeria monocytogenes*.

Hami melon (also known as cantaloupe) is a type of muskmelon, well known for its aesthetic appearance and sweet flavor. This melon is considered the “king of melons” [2]. Due to its richness in nutrients and good taste, Hami melon is a popular fruit item used to make fresh-cut fruits. However, mechanical damage during fresh-cut processing often destroys the tissues of Hami melon, leading to the loss of nutrients and aromatic odor. Furthermore, mechanical damage also facilitates microbial growth, speeding the process of fruit degradation [3]. In addition, the high sugar content of Hami melon further accelerates the growth of microorganisms, which greatly shortens its shelf life. Besides this, long storage periods will also cause fresh-cut fruits to lose water content and become brownish in color, degrading the taste [4]. 

Presently, a number of approaches including coating [5], ultra-violet and violet-blue light irradiation [6,7,8], modified atmosphere packaging [9], and chlorine dioxide treatment [10] have been developed for preserving fresh-cut fruits, but these processes are relatively expensive. Therefore, developing novel approaches that are cheaper alternatives for fresh-cut fruit preservation has great potential in the food industry [11]. 

Photodynamic sterilization technology (PDS) is an environmentally friendly, non-thermal sterilization method. The underlying mechanism involves photosensitizers being activated by lights of certain wavelengths and absorbing the energy of photons to produce reactive oxygen species. These active substances then kill pathogenic microorganisms without damaging the adjacent tissues and cells. PDS was originally used for treating malignant tumors [12], papillomatosis [13], endodontic infection [14], and blood product disinfection [15], and then successfully applied to foods in the 1980s [16,17]. Compared to traditional sterilization technology, PDS possesses a stronger germicidal effect and is less expensive. In addition, this novel technology may also preserve the respective color, fragrance, and freshness of the foods. 

Currently, a number of novel photosensitizers have been identified or synthesized, including both antimicrobial and anti-cancer photosensitizers [18]. Among these photosensitizers, curcumin, a natural plant phenolic food additive, is widely used as a photosensitizer in PDS due to its low cost and safety [17,19]. Studies have already shown that the photodynamic action of curcumin can effectively kill malignant tumor cells [20,21,22]. Recently, scientists also demonstrated curcumin as a photosensitizer that can kill a range of pathogenic microorganisms in food even at low curcumin concentration (25–100 μmol/L) [23,24,25,26]. Furthermore, this method has already shown great potential in the preservation of fresh-cut fruits. For example, curcumin-mediated photodynamic sterilization was successfully applied in the preservation of fresh-cut “Fuji” apples [27]. Therefore, in this study, curcumin-mediated photodynamic treatment was conducted to determine its potential application for the preservation of fresh-cut Hami melon during storage at 4 °C.

## 2. Results and Discussion

### 2.1. Antibacterial Activity of PDS Treatment

The effects of photosensitizer concentration and exposure time on the antibacterial activity of curcumin-mediated PDS for fresh-cut Hami melon preservation are shown in Table 1 and Table 2. The data showed that the bacterial count of the fresh-cut Hami melon markedly increased with storage time. Meanwhile, the curcumin-mediated photodynamic treatment significantly dampened the bacterial growth compared to the control group (*p* < 0.05) (Table 1 and Table 2). Illumination with 50 μmol/L curcumin decreased the bacterial count in the fresh-cut Hami melon from 3.97 (log CFU/g) in the control group to 2.59 (log CFU/g) at 1 day post-treatment. This effect was still obvious till the ninth day of storage as evidenced by the decrease in the bacterial count from 6.81 (log CFU/g) in the control group to 4.95 (log CFU/g) in the fresh-cut Hami melon treated with 50 μmol/L curcumin and blue light, indicating that PDS treatment resulted in ~1.8 log CFU/g reduction when compared to the control after 9 days of storage (Table 1). These results showed that curcumin as a photosensitizer can effectively inhibit bacterial growth in fresh-cut Hami melon. Indeed, our results here are consistent with a previous report in which 2 μmol/L of curcumin and 510 s of illumination was demonstrated to reduce *E. coli* counts by 0.95 logs at 4 °C in fresh-cut apple [27]. 

Hami melon samples sprayed with curcumin solution at 50 μmol/L and illuminated for different time periods were next analyzed for the photodynamic antibacterial effect. Generally, the germicidal efficacy increased with prolonged exposure time (Table 2), but beyond 60 min, no further significant increases were obtained. Notably, even the shortest illumination time in our study (5 min) already led to a significant decrease in the bacterial count compared to the control group (*p* < 0.05). Therefore, the optimal illumination time was chosen as 60 minutes, which was used throughout this study. In a previous study, similar findings were observed that the bacterial killing effect of PDS was more pronounced with prolonged illumination time but leveled off when irradiation time was further increased [28]. 

### 2.2. Effects on Soluble Solids Content

**Figure 1:**
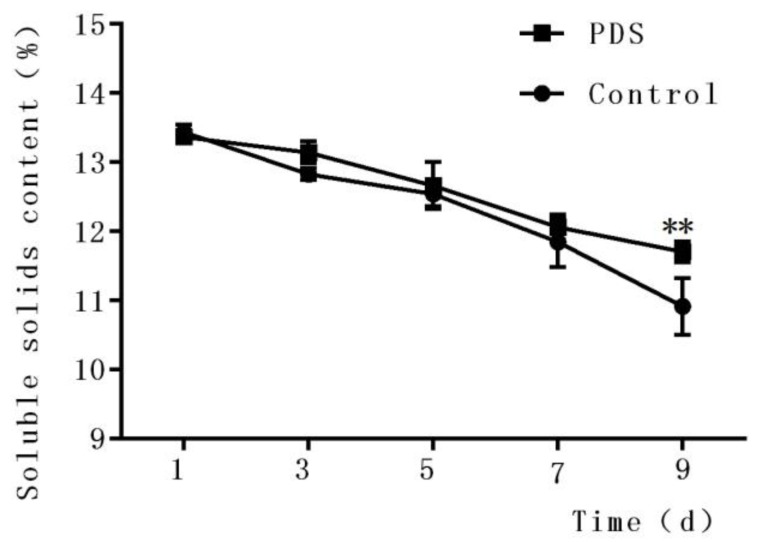
Effects of photodynamic sterilization (PDS) technology on the content of soluble solids in fresh-cut Hami melons. The PDS conditions included 50 μmol/L curcumin and 60 min of illumination with LED light. **p < 0.01 compared to the control group without photosensitizer and light treatment. Data points represent the mean and standard deviation of three experiments.

**Figure 2:**
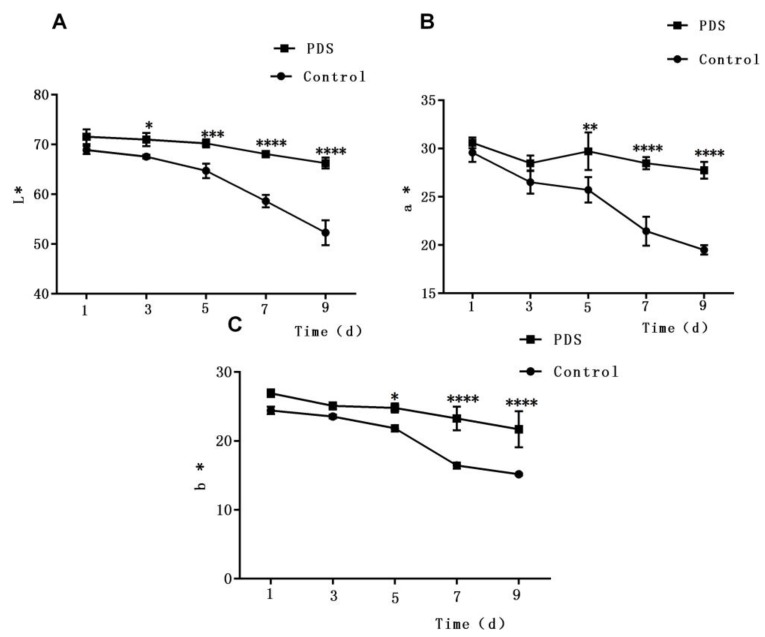
Effect of photodynamic sterilization technology on the L* (lightness) value (**A**), a* (redness) value (**B**), and b* (yellowness) value (**C**) of fresh-cut Hami melons. The PDS conditions used were 50 μmol/L curcumin and 60 min exposure to LED light. *p < 0.05, **p < 0.01, ***p < 0.0005, ****p < 0.0001 compared to the control group without photosensitizer and light treatment. Data points represent the mean and standard deviation of three experiments.

The soluble solid content is an important quality indicator of fresh-cut fruits during storage [29] and is closely associated with the flavor, taste, and nutrition of the fruits. As shown in Figure 1, the content of soluble solids in fresh-cut Hami melon decreased with increasing storage time in both the control and photodynamic-treated Hami melon samples (Figure 2). In the control group, the contents of soluble solids were 13.44%, 12.83%, 12.54%, 11.84%, and 10.92% after 1 day, 3 days, 5 days, 7 days, and 9 days of storage, respectively. The indices of the contents of the soluble solids in the PDS-treated group after 1 day, 3 days, 5 days, 7 days, and 9 days of storage were 13.37%, 13.14%, 12.66%, 12.06%, and 11.71%, respectively. The contents of the soluble solids included sugar, acid, vitamins, and minerals which were influenced by a number of factors, including microbial metabolic activities [30]. Figure 1 indicates that PDS significantly changed the content of soluble solids compared to the control group only at Day 9 (*p* < 0.05). It is possible that microbial respiration affected by PDS, especially during long-term storage of the fruit, contributed to this phenomenon. Indeed, a previous study also demonstrated that applying an antimicrobial coating on fresh-cut mango not only inhibited the growth of microorganisms but also slowed down the decrease of the soluble solids content during storage [31].

### 2.3. Effects on Color

Color is also an important characteristic which determines the sensory quality of fresh-cut fruits [29,32]. To quantify the color changes of fresh-cut Hami melon samples, the L*a*b* coordinates were measured, in which the L* value is the lightness of fruit, while the a* and b* values represent the redness and yellowness, respectively. As shown in Figure 2A, the L* values of the Hami melon samples decreased slowly during storage due to enzymatic browning on the cut surface. Besides this, the L* values of samples with PDS treatment were slightly higher than those of the control group. Furthermore, the downward trend with a sharper slope in samples without PDS suggests that PDS can slow the reduction of the L* value in fresh-cut Hami melon. A similar phenomenon was also reported in a previous study in which results showed that PDS treatment may not only inhibit bacterial growth but also show anti-browning effects and prevent changes in the surface color of fresh-cut apple [27]. Besides this, the a* (redness) value and b* values (yellowness) of the fresh-cut Hami melon showed a similar pattern during storage, in which both decreased sharply after the fifth day (Figure 2B,C). Additionally, significant differences were also observed in the a* and b* values between the treated and control samples after 5 days of storage (*p* < 0.05). Taken together, these results demonstrate that PDS can effectively maintain the surface color of fresh-cut Hami melon throughout the storage period.

### 2.4. Effects on the Water Content

**Figure 3:**
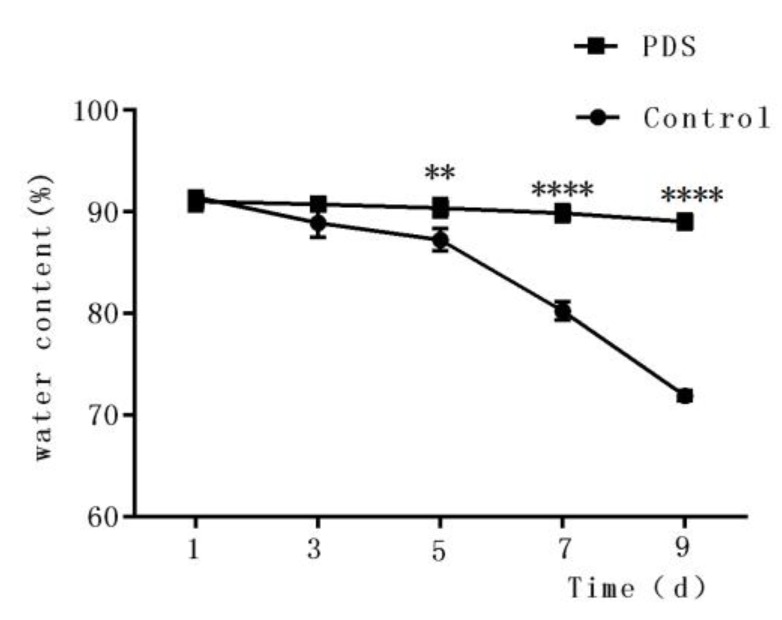
Effects of photodynamic sterilization technology on the water content of fresh-cut Hami melons. The PDS conditions used were 50 μmol/L curcumin and 60 min exposure to LED light. **p < 0.01, ****p < 0.0001 compared to the control group without photosensitizer and light treatment. Data points represent the mean and standard deviation of three experiments.

The water content of fresh-cut fruits is an important factor that influences the sensory quality and consumer perception [30]. In fact, it also affects the physiological metabolism of the fresh-cut fruits, in addition to making fresh-cut Hami melon stiff and full. As shown in Figure 3, the water content decreased under storage conditions. After 9 days of storage, the water contents decreased to 71.93% and 89.04% of the initial moisture content in the control and PDS-treated groups, respectively. The higher water content percentage in the photodynamically sterilized fresh-cut Hami melon might also result from, at least partially, the antibacterial effects of PDS. This can be explained by the fact that rapid microbial growth can result in reduced water content of the fruit. Similar results were also found by Tao et al., who reported that fresh-cut apple with PDS treatment showed less water loss during storage time compared to control samples [27]. These results suggest that PDS treatment may contribute to the maintenance of the water content in fresh-cut Hami melons during their storage.

### 2.5. Effects on the Firmness

**Figure 4:**
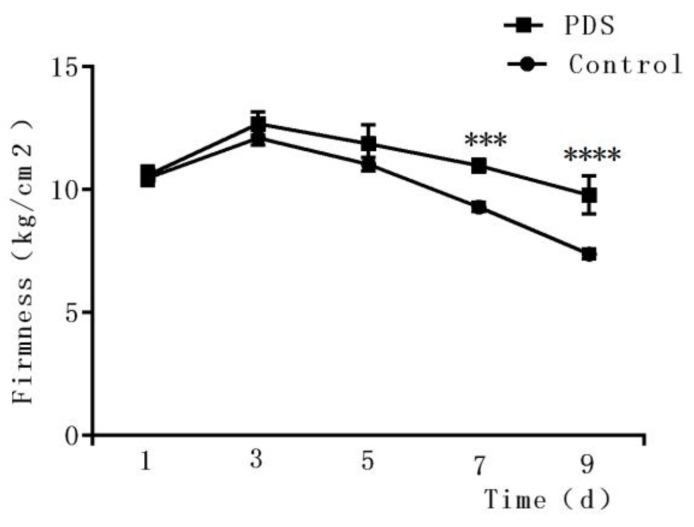
Effects of photodynamic sterilization technology on the firmness of fresh-cut Hami melons. The PDS conditions used were 50 μmol/L curcumin and 60 min exposure to LED light. ***p < 0.0005, ****p < 0.0001 compared to the control group without photosensitizer and light treatment. Data points represent the mean and standard deviation of three experiments.

Firmness is an important parameter reflecting the texture of a fruit [29,32] and may be affected by the ripening, softening, and aging of the fruits [33]. As shown in Figure 4, during the first three days, an upward trend was observed in the firmness of the fresh-cut Hami melon, which may be due to an increase in toughness during storage. Then, the firmness of the fresh-cut Hami melon decreased with increasing storage duration. Several possible mechanisms may be related to this phenomenon, including the rapid growth of microorganisms which convert starch into soluble sugars, transforming the protopectin and pectin into pectic acids and destroying the spatial structure of Hami melon cells [34]. In addition, water loss may also cause the softening of Hami melon on the surface. Here, we also observed that the rate of decrease in firmness in the control group was significantly higher than that in the PDS group. A significant difference can be observed on the ninth day between the PDS-treated and control groups. The firmness in the PDS group was 9.79% higher than that in the control group (*p* < 0.05), suggesting that PDS can delay the decline of firmness in Hami melon. Indeed, a previous study demonstrated that 1-methylcyclopropene treatment inhibited the growth of microorganisms in fresh-cut Hami melon and suppressed its softening during storage [35], suggesting that slowing down the deterioration of fresh-cut Hami melon by inhibiting microorganisms may help to maintain its firmness.

### 2.6. Effects on the Sensory Indices

**Figure 5:**
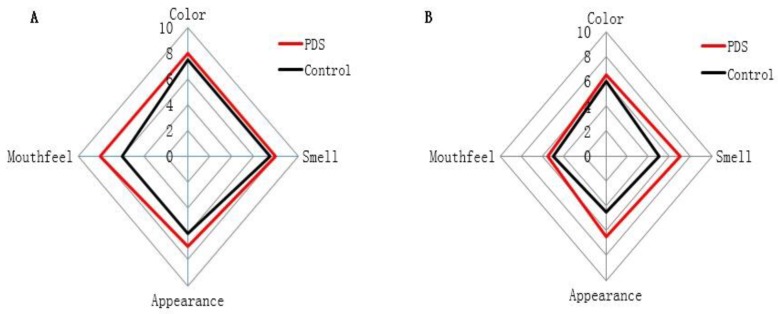
Effect of photodynamic sterilization technology on the sensory indices of fresh-cut Hami melons. The PDS conditions used were 50 μmol/L curcumin and 60 min exposure to LED light. (**A**) The sensory evaluation scores of fresh-cut Hami melon samples in the control groups and photodynamic treatment group after five days of storage. (**B**) The sensory evaluation scores of fresh-cut Hami melon samples in the control groups and photodynamic treatment group after nine days of storage.

The sensory qualities of fresh-cut Hami melon, including color, smell, appearance, and mouthfeel (Table 3), were evaluated in the PDS-treated and control groups during storage (Figure 5). On the fifth day, the color and mouthfeel scores of the fresh-cut Hami melon were not significantly different between the two groups, while the scores of appearance and smell were significantly higher in the PDS-treated group than those in the control group (Figure 5A), and no samples were rotten across these two groups. With the increase in the storage time, the sensory indices of the fresh-cut Hami melon were significantly decreased (Figure 5B). On the ninth day, the scores of the smell and appearance of the fresh-cut Hami melon were higher in the PDS group than those in the control group (Figure 5B). In the control group, visible plaque appeared in the Hami melon, and the surface was softened and rotten, which decreased the quality of the Hami melon. Indeed, a number of studies have suggested that application of PDS treatment in food preservation can result in higher sensory scores upon storage [27,36]. In summary, photodynamic sterilization can effectively delay the deterioration of fresh-cut Hami melon.

## 3. Materials and Methods

### 3.1. Preparation of Fresh-Cut Hami Melon

Ripe Hami melon (~3 kg) in pest-free condition was washed with water and disinfected with 0.21% sodium hypochlorite, followed by rinsing in distilled water. The Hami melon was peeled and then cut into 16 parts on a HEPA clean bench, placed into plastic boxes, and stored at 4 °C prior to PDS treatment within 2 h.

### 3.2. Photosensitizer and Light Source

Curcumin was purchased from Hefei Bomei Biotechnology Co., Ltd. (Anhui, China). A curcumin stock solution was prepared at the concentration of 10 mmol/L. A blue light-emitting diode with a maximum irradiation wavelength of 460 nm was used as light source in all PDS treatments.

### 3.3. Photosensitizer and Irradiation

Fresh-cut Hami melon was sprayed with curcumin solution at different concentrations (10–50 μmol/L) and then exposed to LED illumination for 60 min at room temperature. Saline was used instead of curcumin for the treatment of Hami melon in the control group. For the 50 μmol/L concentration of curcumin, the effect of LED illumination time (5 min, 30 min, 60 min, and 90 min) on the fresh-cut Hami melon was also evaluated. Then, the fresh-cut Hami melon was stored in the fridge at 4 °C.

### 3.4. Microbiological Analyses

The quantification of microorganisms (mainly live, aerobic bacteria) in the fresh-cut Hami melon was performed by the plate colony counting method according to a previous report [37]. Briefly, the samples (~5 g) were homogenized in 100 mL sterile water, sequentially tenfold diluted, and inoculated on the bacteriological substrate plate count agar (PCA). After incubating for 48 h at 37 °C, the number of colonies was counted. The total number of surviving colonies on the plate was counted.

### 3.5. Quality Characteristics and Sensory Evaluation

The soluble solids of the fresh-cut Hami melon were evaluated using a WZ Hand-held saccharometer (Chennuo Biotechnology Co., Ltd., Shanghai, China) according to the manufacturer’s instruction. The color of the fresh-cut Hami melon was measured using an ADCI full-automatic color difference instrument (ADCI, Beijing Chen Taike Instrument Technology Co., Ltd., Beijing, China). The changes of color were represented by values of L* (lightness from black (0) to white (100)), a* (redness from green (-) to red (+)), and b* (yellowness blue (-) to yellow (+)). The moisture content of the fresh-cut Hami melon was determined using an SFY-20A Halogen Rapid Moisture Analyzer (Shenzhen Guanya Electronic Technology Co., Ltd., Shenzhen, China). The firmness of the fresh-cut Hami melon was tested using a TA.XTplus texture apparatus equipped with a P5 cylindrical probe (Beijing Chen Taike Instrument Technology Co., Ltd.). The pre-test speed, test speed, and post-test speed was set to 5 mm/s, 2 mm/s, and 2 mm/s, respectively. The nine-point evaluation method was used to evaluate the sensory scores, including the aroma, taste, and appearance of the fresh-cut Hami melon (Table 3).

### 3.6. Statistical Analysis

Results are presented as the mean ± SEM for at least three independent experiments. Statistical significance was determined using Student’s *t*-test when comparing two groups or using one-way ANOVA followed by post hoc Tukey’s multiple comparisons when comparing three or more groups. The difference between the means was considered statistically significant when *p* < 0.05. All statistical calculations were performed using GraphPad Prism 5.

## 4. Conclusions

Curcumin can be used as a photosensitizer in photodynamic sterilization for food preservation. Here, our results also showed that curcumin-mediated PDS can effectively sterilize residual bacterial contamination in fresh-cut Hami melon and delay their growth. The antibacterial property of the photodynamic sterilization was affected by the length of time of LED exposure and the curcumin concentration. The results showed that microbes in fresh-cut Hami melon could be effectively killed by 50 μ mol /L of curcumin under exposure to the light from a blue LED lamp for 60 min. A previous study demonstrated that in fresh-cut fruit with a neutral pH, such as Hami melon (cantaloupe), bacteria are the main source of spoilage [38]. Therefore, the effects of PDS in preserving the fresh-cut Hami melon in our study may be largely due to its bactericidal activity. However, we cannot rule out that fungicidal activity may also be involved in this process. Furthermore, compared to the control group, color changes were also inhibited effectively by photodynamic sterilization. The firmness, water content, and soluble solid content in the fruits were more stable in the PDS-treated fresh-cut Hami melon. In addition, the sensory qualities of the fresh-cut Hami melon were effectively maintained. Therefore, photodynamic sterilization may be a promising technology that can be utilized conveniently for the preservation of fresh-cut Hami melon and of other fresh-cut fruits in industry.

## Figures and Tables

**Table 1:** Effects of photosensitizer curcumin concentrations on the antibacterial activity of curcumin-based photodynamic sterilization for the preservation of fresh-cut Hami melons.

Storage (day)	Bacterial Count (log CFU/g)				
	Curcumin Concentration				
	0 μmol/L	10 μmol/L	20 μmol/L	40 μmol/L	50 μmol/L
1	3.97 ± 0.04 ^a^	3.76 ± 0.01 ^b^	3.04 ± 0.11 ^d^	3.26 ± 0.05 ^c^	2.59 ± 0.09 ^e^
3	4.99 ± 0.02 ^a^	4.94 ± 0.02 ^a^	4.91 ± 0.09 ^b^	4.67 ± 0.01 ^c^	3.81 ± 0.01 ^d^
5	5.76 ± 0.01 ^a^	5.38 ± 0.09 ^b^	5.23 ± 0.07 ^c^	5.00 ± 0.08 ^d^	4.23 ± 0.03 ^e^
7	6.48 ± 0.07 ^a^	5.91 ± 0.04 ^b^	5.75 ± 0.02 ^b^	5.04 ± 0.03 ^c^	4.77 ± 0.04 ^c^
9	6.81 ± 0.04 ^a^	6.20 ± 0.08 ^b^	5.87 ± 0.05 ^c^	5.11 ± 0.07 ^c^	4.95 ± 0.02 ^c^

Note: The data in the table indicate the mean ± standard deviation, and significant differences exist among the data with different superscript letters in the same row (*p* < 0.05).

**Table 2:** Effects of different exposure time on the antibacterial activity of curcumin-based photodynamic sterilization for the preservation of fresh-cut Hami melons.

Storage (day)	Bacterial Count (log CFU/g)				
	Exposure Time				
	0 min	5 min	30 min	60 min	90 min
1	3.54 ± 0.03 ^a^	3.45 ± 0.08 ^b^	3.28 ± 0.01 ^c^	2.65 ± 0.04 ^d^	2.68 ± 0.06 ^d^
3	4.98 ± 0.02 ^a^	4.26 ± 0.01 ^b^	4.00 ± 0.08 ^c^	3.67 ± 0.02 ^d^	3.68 ± 0.01 ^d^
5	5.83 ± 0.05 ^a^	5.32 ± 0.08 ^b^	4.94 ± 0.12 ^c^	4.20 ± 0.01 ^c^	4.26 ± 0.09 ^c^
7	6.69 ± 0.10 ^a^	5.99 ± 0.11 ^b^	5.40 ± 0.01 ^c^	4.61 ± 0.07 ^d^	4.65 ± 0.08 ^d^
9	6.72 ± 0.07 ^a^	6.11 ± 0.10 ^b^	5.90 ± 0.01 ^b^	4.86 ± 0.08 ^c^	4.90 ± 0.01 ^c^

Note: The data in the table indicate the mean ± standard deviation, and significant differences exist among the data with different superscript letters in the same row (*p* < 0.05).

**Table 3:** Sensory and quality evaluation of fresh-cut Hami melon.

Score	Color	Flavor and Aroma	Surface	Feel
9	Bright	Characteristic aroma	Fresh, plaque-free, and no softening.	The flesh is firm, sweet, crispy, and juicy.
7	Slightly	Normal	Fresh, no plaque, and no softening, slight surface water loss.	The pulp is more compact, harder, and crispy with a thicker sweetness.
5	Normal	Scented	Lack of freshness, obvious surface water loss, and softening.	The sweet taste is light, the pulp is soft, less juice, and not crispy.
3	Dim	Loss of aroma	Local diseased plaque and touch with a slimy feeling.	Not edible.
1	Black	Fermentative, acidic, and rotten	More plaque and more serious softening.	Not edible.
